# Around the Hospitals

**Published:** 1894-08-11

**Authors:** 


### Around the Hospitals.

[Notice to the Managers of Institutions.—Special space is now reserved for the insertion of notices of hospital meetings and festivals. The Editor requests that all notices may reach The Hospital Office, 428, Strand, at least one week before the date of each meeting.]

**The Royal Free Hospital.**—The principal event at the Royal Free Hospital during the past year has been the commencement of the new buildings. The buildings were commenced with a very small part of the necessary funds in hand. Lord Dufferin, as president, made a special appeal through the press, and the contributions which have been received now leave under £7,000 to be collected to complete the work. The hospital has been especially fortunate in the legacies that have fallen to its share. Firstly, it is one of the institutions benefiting under Mr. Spicer's will, and receives thereby £10,500. This sum is to be devoted to the samaritan branch of the work. From Mr. George Fourmer's will £8,000 was received, about £1,500 from Mr. J. H. Valloton, and handsome sums from several others. The income of the hospital is very far behind its requirements, and consequently these legacies have in part to be devoted to current expenses. During the past year a decrease in annual subscriptions was visible. Some generous donations were however received, amongst these being £1,000 from Mrs. Webb, mother of one of the lady students, for the purpose of endowing a bed. Baron de Hirsch contributed £700 to the building fund, and Mr. John Bentley, the Clothworkers' Company, and the Great Northern Railway Company presented sums of £100 and upwards. The expenditure for the year amounted to £10,103, and the receipts to £14,431, including legacies unhampered by conditions, except in the case of £1,000 given for the endowment of a bed. The number of patients was redneed during 1893, as one ward was closed for the rebuilding of the front of the hospital. The in-patients numbered 1,554, the out-patients 26,720. Great care is taken to control expenditure, and the cost per patient amounts to £1 7s. 9d. per week. One special feature at the Royal Free is the admission of lady students in connection with the London School of Medicinc for Women. These now number 160, many of whom have attained distinction in examinations. Another feature in the management of the hospital is the good arrangements for protection in case of fire, and the special drill which nurses and staff of the hospital undergo. Two fires occurred in the institution during the past year, when the excellent arrangements were tested and the fires promptly brought under, and no patients were injured.

**The Bideford Infirmary.**—This is a small hospital, but, besides doing a good work, its system of management contains a feature which should commend itself to both large and small institutions. The system we allude to is that of requiring the patient to provide information as to his means, instead of this being left to the discretion of the recommender or to after inquiry. Thus Rule 14 of the dispensary pro vides that "All applicants must have their weekly earnings, or that of their parents, stated on the recommendation as not exceeding 18s. weekly, or relief will not be given." The number of in-patients benefited during the year amounted to 140, and out-patients to 627. The income for 1893 was £565, and the expenditure £392, with £200 placed on deposit.

**The North-West London Hospital.**—This small hospital, which is the only institution in the midst of the crowded population of the north-west district, echoes the too frequent cry of a decrease in funds. That its sphere of work is a busy one may be guessed from the fact that 17,680 new cases were treated during 1893. The hospital has sustained a heavy loss in the death of the late much esteemed chairman, the Rev. F. J. Ponsonby, in February, 1894. Other deaths among subscribers have tended towards the lowering of contributions, though the committee acknowledge at the same time much generous help received from many benefactors, amongst whom Baron Hirsch should be mentioned as having presented a munificent gift of £700. Like so many other hospitals, it is felt that increased facilities for sending the patients to convalescent homes are sadly needed. During the past year the committee have been able to make better arrangements for the accommodation of the nursing staff, having secured the lease of an adjoining house in Kentish Town Road, and thus added materially to the comfort of nurses and patients. An urgent appeal for help concludes the report for the last year, and all who wish to judge for themselves of the work of the hospital are earnestly invited to make a personal visit of inspection.

